# GAPT regulates cholinergic dysfunction and oxidative stress in the brains of learning and memory impairment mice induced by scopolamine

**DOI:** 10.1002/brb3.1602

**Published:** 2020-03-15

**Authors:** Zhenhong Liu, Gaofeng Qin, Lulu Mana, Yunfang Dong, Shuaiyang Huang, Yahan Wang, Yiqiong Wu, Jing Shi, Jinzhou Tian, Pengwen Wang

**Affiliations:** ^1^ Key Laboratory of Chinese Internal Medicine of Ministry of Education and Beijing Dongzhimen Hospital Beijing University of Chinese Medicine (BUCM) Beijing China; ^2^ Department of Integrative Medicine School of TCM Xinjiang Medical University Urumqi China; ^3^ Zhongkang International Health Physical Examination Center‐Qingdao Ruiyuan Hospital of Traditional Chinese Medicine Qingdao China; ^4^ Jiangsu Province Hospital on Integrated Chinese and Western Medicines Nanjing China; ^5^ BUCM Neurology Center Dongzhimen Hospital Beijing University of Chinese Medicine Beijing China

**Keywords:** Alzheimer's disease, behavior, cholinergic system, GAPT, memory impairment, oxidative stress, scopolamine

## Abstract

**Background:**

Cholinergic dysfunction and oxidative stress are the crucial mechanisms of Alzheimer's disease (AD). GAPT, also called GEPT (a combination of several active components extracted from the Chinese herbs ginseng, epimedium, polygala and tuber curcumae) or Jinsiwei, is a patented Chinese herbal compound, has been clinically widely used to improve learning and memory impairment, but whether it can play a neuroprotective role by protecting cholinergic neurons and reducing oxidative stress injury remains unclear.

**Methods:**

Male ICR mice were intraperitoneally injected with scopolamine (3 mg/kg) to establish a learning and memory disordered model. An LC‐MS method was established to study the chemical compounds and in vivo metabolites of GAPT. After scopolamine injection, a step‐down passive‐avoidance test (SDPA) and a Y maze test were used to estimate learning ability and cognitive function. In addition, ELISA detected the enzymatic activities of acetylcholinesterase (AChE), acetylcholine (ACh), choline acetyltransferase (ChAT), malondialdehyde (MDA), glutathione peroxidase (GPX), and total superoxide dismutase (T‐SOD). The protein expressions of AChE, ChAT, SOD1, and GPX1 were observed by western blot, and the distribution of ChAT, SOD1, and GPX1 was observed by immunohistochemical staining.

**Results:**

After one‐half or 1 month of intragastric administration, GAPT can ameliorate scopolamine‐induced behavioral changes in learning and memory impaired mice. It can also decrease the activity of MDA and protein expression level of AChE, increase the activity of Ach, and increase activity and protein expression level of ChAT, SOD, and GPX in scopolamine‐treated mice. After one and a half month of intragastric administration of GAPT, echinacoside, salvianolic acid A, ginsenoside Rb1, ginsenoside Rg2, pachymic acid, and beta asarone could be absorbed into mice blood and pass through BBB.

**Conclusions:**

GAPT can improve the learning and memory ability of scopolamine‐induced mice, and its mechanism may be related to protecting cholinergic neurons and reducing oxidative stress injury.

## Graphical abstract

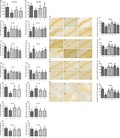

## 1 INTRODUCTION

AD is thought to be a neurodegenerative disease. The prominent features of AD are the impairment of memory and cognition (Lane, Hardy, & Schott, 2018) and neuropathological hallmarks such as loss of neurons and synapses (Goedert & Spillantini, 2006), the agglomeration of neuritic plaques in the cortex (Grösgen, Grimm, Friess, & Hartmann, 2010), and the presence of intracellular neurofibrillary tangles (NFTs). Several hypotheses have been proposed in earlier studies. However, the mechanisms underlying AD are quite complicated and still uncertain. Some studies have noted that oxidative stress and cholinergic dysfunction play a critical role in the progression of AD (Kim et al., 2019).

To improve the research about cognitive impairments in AD, many mouse models and behavioral tests have been developed. Scopolamine (a nonselective muscarinic blocker) has been widely used to establish an AD‐like model since it can induce cognitive and memory deficits by promoting acetylcholinesterase (AChE) and upregulating brain iron (Wang, Zhong, Gao, & Li, 2017).

Acetylcholine (ACh) and cholinergic nerves existing in the hippocampus and cortex are essential for regulation of learning and memory processes (Orta‐Salazar, Cuellar‐Lemus, Díaz‐Cintra, & Feria‐Velasco, 2014). The neurotransmitter ACh is employed by all cholinergic neurons, and its normal physiological function guarantees the storage and elicitation of memories (Papandreou et al., 2011). The degeneration of neurons, which is closely related to a loss of cholinergic markers, resulting in cholinergic system dysfunction has been thought to be the most consistent changes in AD cases (Hampel et al., 2018). Choline acetyltransferase (ChAT) catalyzes the synthesis of Ach from choline acetyl‐CoA and choline, which is decomposed by AChE (Nalivaeva & Turner, 2016). Some evidence suggests that the increase of AChE is one of the important causes of AD (Lahiri et al., 2002; Racchi, Sironi, Caprera, König, & Govoni, 2001), and currently, clinically available AChE inhibitors for AD, such as donepezil, galantamine, and rivastigmine, can ameliorate the cognitive symptoms and enhance the living quality for AD patients.

Oxidative imbalance and neuronal damage also play a key role in the initiation and progression of AD. In patients with AD, oxidative stress is a state of imbalance between ROS production and antioxidant defense, leading to excessive accumulation of ROS (Tönnies & Trushina, 2017). Excessive ROS in the body can peroxidate unsaturated fatty acids on the cell membrane of neurons in the brain and form lipid peroxidation products. MDA is one of the products of lipid peroxidation. The accumulation of MDA will destroy the cell membrane structure and eventually lead to cell damage and even death. SOD and GPX are the main antioxidant enzymes in the body, which can reduce the memory impairment and cell damage induced by oxidative stress, and jointly remove excessive ROS to maintain the homeostasis between oxidation and antioxidant in the body (Ferreira et al., 2015).

In the treatment of AD, multitarget and multichannel combined treatment has attracted great attention. It is worth mentioning that there are some remarkable traditional Chinese medicine compounds that have been gradually applied in clinical treatment of learning and memory impairment for their multitarget therapeutic effects. Among them, GAPT, also called as GEPT or GETO in our previous papers, is a combination of herbal extracts and a patented Chinese herbal compound for AD. GAPT, composed of 4.4% ginsenoside from ginseng, 17.3% cistanche, 17.3% prepared Radix Rehmanniae, 13% processed *Polygala tenuifolia*, 13% *Acorus gramineus*, 13% Wide Radix Curcumae, 13% Poria cocos, and 9% *Salvia officinalis* (Shi et al., 2016; Tian et al., 2009), has been clinically widely used to improve learning and memory impairment. A single‐blind, randomized controlled clinical trial shows that 75 patients with suspected dementia were treated with GAPT for 3 months, and 1‐year follow‐up showed that GAPT can significantly improve the memory test scores compared with placebo group (Tian, Zhu, & Zhong, 2003). In another study, GAPT can effectively reduce GSK‐3β expression level in the brain cortex of APPV7171 transgenic mice, thus playing a neuroprotective role (Shi et al., 2013). It also regulates the expression of CDK5 and PP2A in hippocampal neurons, thereby inhibiting abnormal tau phosphorylation (Ni et al., 2017). GAPT can increase APP/PS1 transgenic mice's brain glucose uptake and glucose transport and improve the insulin signaling pathway (Mana et al., 2019). Moreover, synapse damage ameliorated by GAPT via regulating bcl‐2/Bax balance (Shi et al., 2018).

While the mechanisms behind protecting cholinergic neurons and reducing oxidative stress of GAPT remain unclear, we hypothesized that GAPT can improve the cognitive ability of the scopolamine‐induced AD‐like mice. We also studied the pharmacodynamics of different doses of GAPT. This study will investigate the optimal dose of GAPT for preventing and treating learning and memory disorder and further explore the neuroprotective mechanism of GAPT from cholinergic system and oxidative stress, thus providing the theoretical basis for the better application of GAPT in clinical practice.

## 2 MATERIALS AND METHODS

### 2.1 Drugs preparation

GAPT, a patented Chinese herbal compound (Patent NO. ZL200810006733.0), was purchased from Henan Wanxi Pharmaceutical Company Limited (Batch No: 20010923). A concentration of 30 mg/ml GAPT was configured with 0.5% carboxymethyl cellulose (CMC). Hydrochloric acid donepezil tablets were purchased from Eisai Pharmaceutical Company Limited (Batch No. 140635), and a concentration of 0.092 mg/ml donepezil was configured with 0.5% carboxymethyl cellulose (CMC). Scopolamine was purchased from Harvest Pharmaceutical Company Limited (Batch No. 02161001, Shanghai, China) and configured to 3 mg/kg for intraperitoneal injection. The reference standards of verbascoside (no. 2659/20556), ginsenoside Rb1 (no. 2326/13523), and ginsenoside Re (no. 2070/9407) were obtained from Shanghai Standard Biotech Co., Ltd. Tenuifolin (no. 141205) was obtained from Chengdu Pufei De Biotech Co., Ltd). Salvianolic acid A (no. MUST‐14040401), Salvianolic acid B (no. MUST‐13103113), and ginsenoside Rg2 (no. MUST‐13062113) were obtained from Chengdu Manster Biotech Co., Ltd. Echinacoside (no. B21209), Curcumin (no. B20614), Pachymic acid (no. B20400), and beta asarone (no. B30631) was obtained from Shanghai Yuanye Bio‐Technology Co., Ltd.

### 2.2 Animals and drug administration

This research used 6‐month‐old male ICR mice 28–30 g in weight that purchased from Beijing Huafukang Biotechnology Co., Ltd (SCXK (Beijing) 2014‐0004). The animals are kept in SPF grade animal laboratories in Dongzhimen Hospital affiliated to Beijing university of Chinese medicine (Certificate SYXK2015‐0001, Beijing, China). Animals are given regular gavage in the morning and free food and water during feeding. All experiments were performed in compliance with Beijing's regulations and guidelines for the use of animals in research, and the study was approved by the Animal Research Ethics Board of Dongzhimen Hospital (Approval No. 17‐09).

Animal experiments were divided into two stages. In the first stage, animals were randomly distributed into six groups containing the control group, the model group, the donepezil group, and the low, medium, and high dosage GAPT groups. In the second stage, animals were randomly distributed into four groups containing the control group, the model group, the donepezil group, and the medium dosage GAPT groups. The control group and model group were administered 0.5% CMC, donepezil group mice were treated with donepezil (0.92 mg kg^−1^ day^−1^), and the GAPT groups were administered a small dose (0.405 g kg^−1^ day^−1^), a medium dose (0.81 g kg^−1^ day^−1^), and a large dose (1.62 g kg^−1^ day^−1^) of GAPT for one‐half (first stage) or 1 month (second stage). One and a half hours after intragastric administration, mice were intraperitoneally injected with scopolamine (3 mg/kg, 0.1 ml/10 g). Control groups were administered a 0.9% normal saline injection of the same volume and via the same route.

### 2.3 LC‐MS analysis

Half a month of intragastric administration of GAPT (medium dose), mice plasma, and brain were collected for LC‐MS analysis. The UHPLC separation was carried out using Ultimate 3000 ultra‐high‐performance liquid chromatograph, equipped with a XSelect HSS T3 C18 column (2.1 mm × 75 mm, 2.5 µm). The mobile phase A was acetonitrile, and the mobile phase B was 0.1% formic acid aqueous solution. The elution gradient was shown as follows: (a) 0‐15 min, 5%–50% A, (b) 15–22 min, 50%–95% A, and (c) 22–26 min, 95% A. The column temperature was set at 25°C, and the flow rate was set at 0.40 ml/min.

LTQ‐Orbitrap velos pro mass spectrometer (ESI) was applied for chemical analysis. Typical ion source parameters were as follows: capillary voltage = 35 V, nozzle voltage = 3.4 kV, gas (N_2_) flow = 10 arb, sheath gas (N_2_) flow = 35 arb, capillary temperature = 350°C, and heater temperature = 350°C. Mass standard calibration using external standards method (mass error is less than 5 ppm), the first‐order mass spectrum is scanned in FT mode (resolution R is 30,000, scan range is from 50 to 1,500), and the MS^2^ and MS^3^ are data‐dependent scan. Data acquisition and analysis were performed using Xcalibur 2.1 workstation (Thermo‐Fisher), Metaworks, Mass Frontier 7.0 software.

### 2.4 Step‐down passive‐avoidance test (SDPA)

The step‐down passive‐avoidance test was carried out according to previous experimental procedures (Figueiró et al., 2011; Mana et al., 2019). The experiment was divided into 2 days (1 day for training and another for testing). During the training (the first day), the mice were put into training apparatus (Shanghai Transfer Information Technology CO., LTD) with five connected 150 × 300 × 300 rooms (had copper grids at the bottom of each room), and then, a 36 V current was passed through the bottom of the room. Under normal conditions, the mice were shocked and jumped onto a shock freezone (SFZ) (an insulating platform 4.5 cm high in the middle of each room) to avoid the shock. 24 hr later, the mice were placed on the SFZ. The step‐down latency (SDL) was the time when the mice first jumped off the SFZ, and the number of times the mice jumped off the SFZ within 5 min was the error times (ET).

### 2.5 Y maze test

The Y‐maze is an apparatus with three equal identical black Plexiglas arms (40 × 4.5 × 12 cm, 120° apart), and each arm has a movable partition at the center of the maze. Three arms are randomly distributed into novel arm, a start arm, and another arm. The test contains two phases. First, 10 min after scopolamine injection, mice were placed in the start arm with 3 min free exploring time and one arm blocked. One hour later, mice were free to explore the entire maze for 3 min with the baffle removed. The time and distance in novel arm were recorded and analyzed (Shanghai transfer information technology CO., LTD).

### 2.6 Enzymatic activities of MDA, ACh, AChE, ChAT, SOD, and GPX were determined by ELISA

Mice were anesthetized with 20 mg/ml tribromoethanol and sacrificed by cervical dislocation. We immediately stripped brain tissues (removal of the cerebellum) on ice, rinsed with precooled saline, and dried on filter paper. The tissues were weighed and homogenized with ultrasound in saline, centrifuged at 4°C at 3,000–4,000 r/min, and then, supernatant was taken for detecting the activities of ACh, AChE, ChAT, MDA, GPX, and T‐SOD by employing mouse‐specific ELISA kits (Nanjing Jiancheng Bioengineering Institute, Nanjing, China) according to the manufacturer's protocols.

### 2.7 Western blot analysis

The EP tube containing hippocampal tissue was added with RIPA tissue/cell lysate solution (R0010, Solarbio), homogenized by ultrasonic grinder, and centrifuged to obtain supernatant. The concentration of hippocampal tissue was measured with BCA method. Separation of protein by SDS‐PAGE, electro transfer, antigen and antibody reaction, color rendering, and exposure were performed respectively. Importantly, in immunodetection, the membranes were probed with primary antibodies: AChE (1:2,000, Abcam), ChAT (1:1,000, Abcam), SOD1 (1:2,000, Abcam), GPX1 (1:1,000, Abcam), and β‐actin (1:5,000, Abcam) at 4°C overnight. Finally, the protein bands were analyzed by Image J software, and the gray value of internal reference β‐actin was compared to analyze the results and calculate the relative percentage.

### 2.8 Immunohistochemical Staining

The immunohistochemical staining was performed following the protocol of our previous study (Wang et al., 2019). Embedded paraffin tissue from the brain of mice were made into continuous coronal sections with a thickness of about 4 μm, and then, deparaffinize and rehydrate tissue sections, antigen retrieval (microwave), quenching of endogenous peroxides, blocking, primary antibody incubation, and detection were performed. Finally, three fields of vision per section were selected to observe the CA1 region of the hippocampus under 20× objective lens and the number of positive cells was counted. Simultaneously, The ChAT‐positive cells in basal forebrain were counted under 5× objective lens. Six paraffin sections were taken from each group and analyzed by Image‐Pro Plus image analysis software.

### 2.9 Statistical analysis

The data were expressed as the mean ± *SD*. Statistical analysis and data plotting were performed via one‐way analysis of variance (ANOVA) using SPSS20.0 software and GraphPad Prism 6. Values of *p* < .05 were considered to be significant.

## 3 RESULTS

### 3.1 GAPT‐medicated plasma and brain contained Ginsenoside Rb1, Beta asarone, and other components by LC‐MS analysis

**Figure 1:**
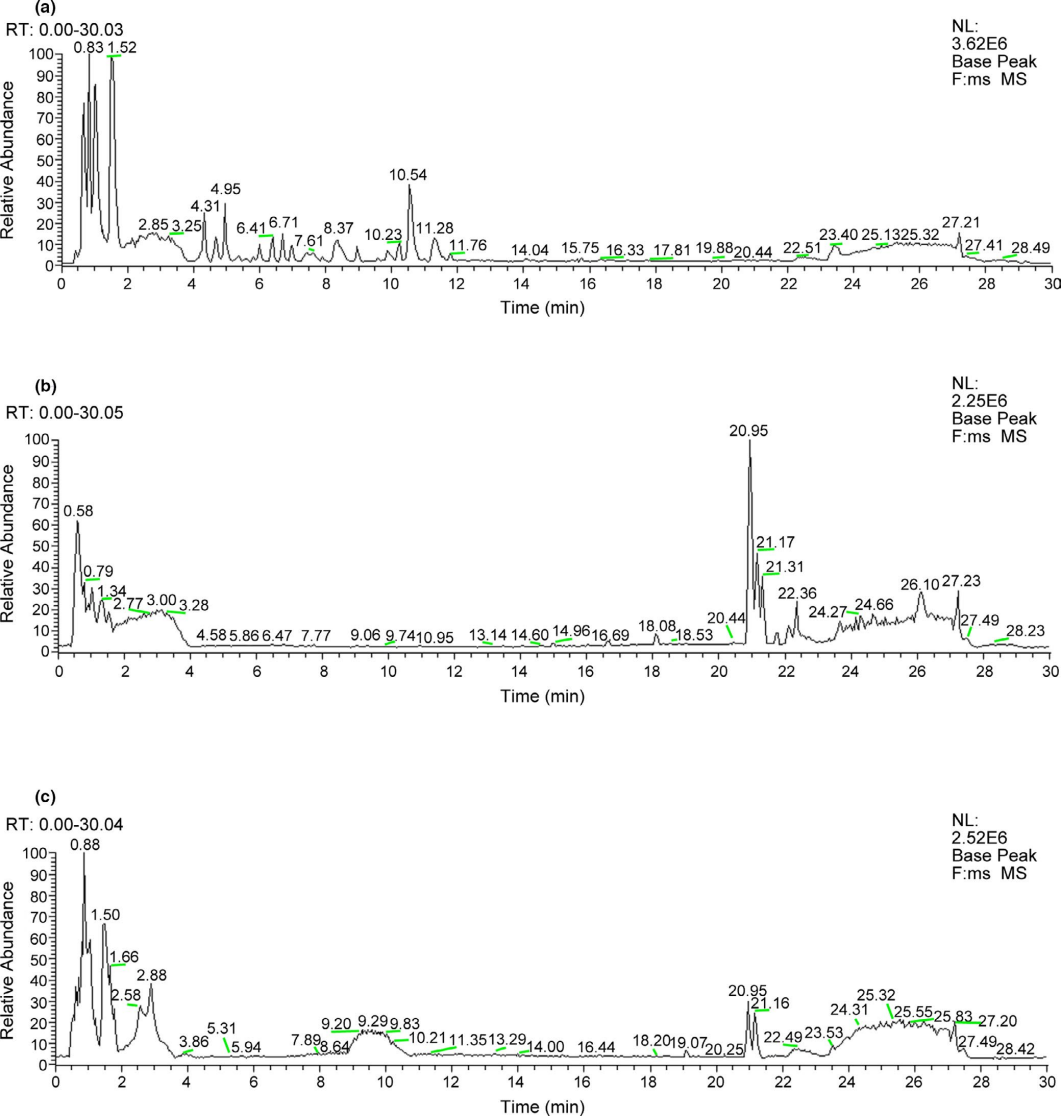
The total ion chromatogram (TIC) of GAPT. (a) GAPT; (b) GAPT‐medicated plasma; (c) GAPT‐medicated brain

First, to confirm the real effects of GAPT in neurons and brain regions, we chose LC‐MS for qualitatively analyzing the components in the GAPT‐medicated plasma and brain. As shown in Figure 1 and Table 1, by LC‐MS analysis, a total of 83 compounds were identified from GAPT, a total of 42 compounds were identified from GAPT‐medicated plasma, and a total of 43 compounds were identified from GAPT‐medicated brain. Among 42 compounds in GAPT‐medicated plasma, 5 compounds (10, 50, 64, 82, and 83) were identified by comparing with reference standards, while the other 37 compounds were characterized based on literatures. At the same time, among 43 compounds in GAPT‐medicated brain, 6 compounds (32, 50, 64, 67, 79, and 82) were identified by comparing with reference standards, while the other 37 compounds were characterized based on literatures.

**Table 1:** Qualitative analysis on chemical compounds in GAPT (*n* = 3)

No.	Ionization mode	*t*_R_ (min)	Measured (m/z)	Predicted (m/z)	Formula	RDB	Error (ppm)	Compound	GAPT	Plasma	Brain	References
1	ESI‐	0.67	665.2119	665.2135	C_24_H_41_O_21_	4	−2.457	Tetrasccharide	√	/	/	Chen et al. (2020)
2	ESI‐	0.67	179.0552	179.0550	C_6_H_11_O_6_	1	1.259	Monosaccharide	√	/	/	Chen et al. (2020)
3	ESI‐	0.67	325.1115	325.1071	C_19_H_17_O_5_	11	13.533	Ailanthoidol	√	√	√	Chen et al. (2020)
4	ESI‐	0.71	267.1061	267.1016	C_17_H_15_O_3_	10	16.994	Danshenspiroketallactone	√	√	√	Chen et al. (2020)
5	ESI‐	0.79	313.0761	313.0707	C_17_H_13_O_6_	11	17.297	Salvianolic acid F	√	/	/	Chen et al. (2020)
6	ESI‐	1.69	191.0223	191.0186	C_6_H_7_O_7_	3	19.008	Citric acid	√	/	√	Chen et al. (2020)
7	ESI‐	2.14	373.1112	373.1129	C_16_H_21_O_10_	6	−4.672	Geniposodic acid	√	√	/	Yan (2018)
8	ESI‐	2.14	799.2690	799.2655	C_36_H_47_O_20_	13	4.329	Cistanoside A	√	√	√	Yan (2018)
9	ESI‐	2.5	717.1496	717.1450	C_36_H_29_O_16_	22	6.399	Salvianolic acid L	√	√	/	Chen et al. (2020)
10	ESI‐	2.5	717.1496	717.1450	C_36_H_29_O_16_	22	6.399	Salvianolic acid B*	√	√	/	/
11	ESI‐	2.5	347.1316	347.1337	C_15_H_23_O_9_	4	−5.959	Leonuride	√	√	√	Zhao, Li, and Sun (2007)
12	ESI‐	2.55	509.1843	509.1865	C_21_H_33_O_14_	5	−4.226	Rehmannioside C	√	/	/	Zhao et al. (2007)
13	ESI‐	2.59	1791.7466	1791.7390	C_72_H_127_O_50_	9	4.253	Onjisaponin T	√	/	√	Liu et al. (2012)
14	ESI‐	2.68	375.1263	375.1286	C_16_H_23_O_10_	5	−6.113	8‐*epi*‐loganic acid	√	√	√	Yan (2018)
15	ESI‐	2.81	345.1161	345.1180	C_15_H_21_O_9_	5	−5.472	Aucubin	√	√	/	Zhao et al. (2007)
16	ESI‐	2.85	537.1043	537.1028	C_27_H_21_O_12_	17	2.9	Alkannic acid	√	/	√	Chen et al. (2020)
17	ESI‐	2.85	537.1043	537.1028	C_27_H_21_O_12_	17	2.9	Salvianolic acid	√	/	√	Chen et al. (2020)
18	ESI‐	3.48	523.1643	523.1657	C_21_H_31_O_15_	6	−2.861	Rehmannioside A/B	√	/	/	Zhao et al. (2007)
19	ESI‐	3.76	517.1514	517.1552	C_22_H_29_O_14_	8	−7.371	Sibiricose A5	√	/	/	Liu et al. (2012)
20	ESI‐	3.96	547.1616	547.1657	C_23_H_31_O_15_	8	−7.542	Sibiricose A6	√	/	/	Liu et al. (2012)
21	ESI‐	3.96	547.1616	547.1657	C_23_H_31_O_15_	8	−7.542	Sibiricose A1	√	/	/	Liu et al. (2012)
22	ESI‐	4.03	475.1783	475.1810	C_21_H_31_O_12_	6	−5.793	Cistanoside E	√	/	√	Yan (2018)
23	ESI‐	4.31	717.1516	717.1450	C_36_H_29_O_16_	22	9.202	Salvianolic acid B isomer	√	√	/	Chen et al. (2020)
24	ESI‐	4.4	375.1282	375.1286	C_16_H_23_O_10_	5	−1.075	8‐*epi*‐loganic acid isomer	√	√	√	Yan (2018)
25	ESI‐	4.67	417.0829	417.0816	C_20_H_17_O_10_	12	2.966	Salvianolic acid D	√	/	√	Chen et al. (2020)
26	ESI‐	4.72	503.1722	503.1759	C_22_H_31_O_13_	7	−7.428	Cistanoside H	√	√	√	Yan (2018)
27	ESI‐	4.82	487.1409	487.1446	C_21_H_27_O_13_	8	−7.692	Cistanoside F isomer	√	/	√	Chen et al. (2020)
28	ESI‐	4.95	197.0441	197.0444	C_9_H_9_O_5_	5	−1.623	Tanshinol	√	√	√	Chen et al. (2020)
29	ESI‐	4.95	487.1465	487.1446	C_21_H_27_O_13_	8	3.762	Cistanoside F	√	/	√	Yan (2018)
30	ESI‐	4.95	639.1876	639.1920	C_29_H_35_O_16_	12	−6.886	Campneoside Ⅱ or lugrandoside	√	/	/	Yan (2018)
31	ESI‐	4.95	785.2463	785.2499	C_35_H_45_O_20_	13	−4.495	Echinacoside isomer	√	/	/	Yan (2018)
32	ESI‐	4.97	786.2520	786.2577	C_35_H_46_O_20_	13	−7.23	Echinacoside*	√	/	√	/
33	ESI‐	4.99	405.0796	405.0816	C_19_H_17_O_10_	11	−5.093	Lancerin	√	/	√	Xu et al. (2016)
34	ESI‐	5.11	767.2330	767.2393	C_35_H_43_O_19_	14	−8.257	Tenuifoliside C	√	/	/	Liu et al. (2012)
35	ESI‐	5.17	537.1203	537.1239	C_24_H_25_O_14_	12	−6.668	Sibiricaxanthone A or B	√	/	/	Liu et al. (2012)
36	ESI‐	5.36	769.2496	769.2550	C_35_H_45_O_19_	13	−6.923	Poliumoside	√	/	/	Yan (2018)
37	ESI‐	5.48	567.1298	567.1344	C_25_H_27_O_15_	12	−8.158	Polygalaxanthone III	√	√	/	Liu et al. (2012)
38	ESI‐	5.61	521.1986	521.2017	C_26_H_33_O_11_	10	−6.117	Lariciresinol−4‐*O*‐*β*‐D‐glucopyranoside	√	√	√	Yan (2018)
39	ESI‐	5.8	345.1523	345.1544	C_16_H_25_O_8_	4	−6.038	Kankanoside A or isomer	√	√	√	Yan (2018)
40	ESI‐	5.86	667.1815	667.1869	C_30_H_35_O_17_	13	−8.118	Tenuifoliside B	√	/	/	Liu et al. (2012)
41	ESI‐	5.86	193.0489	193.0495	C_10_H_9_O_4_	6	−3.446	Ferulic acid	√	√	√	Chen et al. (2020)
42	ESI‐	5.86	193.0489	193.0495	C_10_H_9_O_4_	6	−3.446	Isoferulic acid	√	√	√	Chen et al. (2020)
43	ESI‐	5.99	623.1926	623.1970	C_29_H_35_O_15_	12	−7.183	Verbascoside*	√	/	/	/
44	ESI‐	6.29	519.1821	519.1861	C_26_H_31_O_11_	11	−7.624	Pinoresinol‐*O*‐*β*‐D‐glucopyranoside	√	/	√	Yan (2018)
45	ESI‐	6.41	623.1935	623.1970	C_29_H_35_O_15_	12	−5.627	Isoacteoside	√	/	/	Yan (2018)
46	ESI‐	6.71	753.2174	753.2237	C_34_H_41_O_19_	14	−8.291	3,6’‐Disinapoylsucrose	√	/	/	Liu et al. (2012)
47	ESI‐	6.99	493.1113	493.1129	C_26_H_21_O_10_	16	−3.353	Salvianolic acid A isomer	√	√	√	Chen et al. (2020)
48	ESI‐	6.99	607.1970	607.2021	C_29_H_35_O_14_	12	−8.419	Syringalide A 3′‐*O*‐α‐L‐rhamnopyranoside or isomer	√	√	√	Yan (2018)
49	ESI‐	7.15	368.1264	368.1254	C_21_H_20_O_6_	12	2.527	Curcumin*	√	/	/	/
50	ESI‐	7.34	493.1157	493.1129	C_26_H_21_O_10_	16	5.672	Salvianolic acid A*	√	√	√	/
51	ESI‐	7.34	637.2076	637.2127	C_30_H_37_O_15_	12	−8.03	Cstanoside C	√	√	/	Yan (2018)
52	ESI‐	7.34	637.2076	637.2127	C_30_H_37_O_15_	12	−8.03	Cstanoside C isomer	√	√	/	Yan (2018)
53	ESI‐	7.45	681.1969	681.2025	C_31_H_37_O_17_	13	−8.259	Tenuifoliside A	√	/	/	Liu et al. (2012)
54	ESI‐	7.61	665.2027	665.2076	C_31_H_37_O_16_	13	−7.383	2‐Acetylacteoside	√	√	/	Yan (2018)
55	ESI‐	7.61	665.2027	665.2076	C_31_H_37_O_16_	13	−7.383	Tbuloside B	√	√	/	Yan (2018)
56	ESI‐	7.71	373.1136	373.1129	C_16_H_21_O_10_	6	1.867	Gniposidic acid isomer	√	√	/	Yan (2018)
57	ESI‐	7.77	591.2034	591.2072	C_29_H_35_O_13_	12	−6.508	Osmanthuside B or osmanthuside B6 or isomer	√	√	/	Yan (2018)
58	ESI‐	7.83	651.2230	651.2283	C_31_H_39_O_15_	12	−8.272	Cistanoside D	√	/	/	Yan (2018)
59	ESI‐	7.96	537.0994	537.1028	C_27_H_21_O_12_	17	−6.298	Salvianolic acidH/I	√	/	√	Chen et al. (2020)
60	ESI‐	8.02	329.1365	329.1384	C_19_H_21_O_5_	9	−5.5	13*R*‐14*R*‐hydroxy‐anhydride of 16 Rcryptotanshinone	√	√	√	Chen et al. (2020)
61	ESI‐	8.15	767.2329	767.2393	C_35_H_43_O_19_	14	−8.336	Tenuifoliside C	√	/	/	Liu et al. (2012)
62	ESI‐	9.62	491.0977	491.0973	C_26_H_19_O_10_	17	0.788	Isosalvianolic acid C	√	/	√	Chen et al. (2020)
63	ESI‐	9.67	799.4765	799.4838	C_42_H_71_O_14_	7	−9.172	Ginsenoside Rf	√	/	/	Liu et al. (2012)
64	ESI‐	10.3	1,107.6044	1,107.5946	C_54_H_91_O_23_	9	8.852	Ginsenoside Rb1*	√	√	√	/
65	ESI‐	11.28	991.5408	991.5472	C_49_H_83_O_20_	8	−6.446	Ginsenoside Re*	√	/	/	/
66	ESI‐	12.44	513.3171	513.3211	C_31_H_45_O_6_	9	−7.647	Poricoic acid D	√	√	√	Liu (2004)
67	ESI‐	14.1	783.4815	783.4889	C_42_H_71_O_13_	7	−9.532	Ginsenoside Rg2*	√	/	√	/
68	ESI‐	15.96	285.1475	285.1485	C_18_H_21_O_3_	8	−4.037	Crytoacetalide/ epi‐crytoac‐etalide	√	√	√	Chen et al. (2020)
69	ESI‐	16.02	363.1309	363.1286	C_15_H_23_O_10_	4	6.297	Kankanoside B	√	√	√	Yan (2018)
70	ESI‐	17.39	471.3434	471.3469	C_30_H_47_O_4_	7	−7.312	16α‐Hydroxytrametenolic	√	√	√	Zou, Xu, Long, Zhang, and Li (2019)
71	ESI‐	17.39	483.3433	483.3469	C_31_H_47_O_4_	8	−7.503	3‐EpidehydrotuMulosic acid	√	√	/	Kang, Guo, Xie, Shan, and Di (2014)
72	ESI‐	18.43	481.3279	481.3312	C_31_H_45_O_4_	9	−6.973	Polyporenic acid C	√	/	√	Zou et al. (2019)
73	ESI‐	18.43	717.1447	717.1450	C_36_H_29_O_16_	22	−0.503	Salvianolic acid E	√	√	/	Chen et al. (2020)
74	ESI‐	18.69	485.3229	485.3262	C_30_H_45_O_5_	8	−6.802	Poricoic acid G	√	√	√	Kang et al. (2014)
75	ESI‐	20.11	513.3542	513.3575	C_32_H_49_O_5_	8	−6.352	Poricoic acid HM	√	√	/	Kang et al. (2014)
76	ESI‐	20.36	327.1238	327.1227	C_19_H_19_O_5_	10	3.484	1S‐hydroxy‐anhydride of 16Rcryptotanshinone	√	√	√	Chen et al. (2020)
77	ESI‐	20.44	525.3553	525.3575	C_33_H_49_O_5_	9	−4.114	3‐Epidehydropachymic acid	√	/	√	Kang et al. (2014)
78	ESI‐	20.6	499.3383	499.3418	C_31_H_47_O_5_	8	−6.971	Poricoic acid GM/H	√	√	/	Akihisa et al. (2009)
79	ESI‐	20.74	527.3695	527.3731	C_33_H_51_O_5_	8	−6.81	Pachymic acid*	√	/	√	/
80	ESI‐	24.45	313.1443	313.1434	C_19_H_21_O_4_	9	2.728	Neocryptotanshinone	√	√	√	Chen et al. (2020)
81	ESI‐	26.42	1,337.3938	1,337.3978	C_60_H_73_O_34_	24	−2.972	Tenuifoliose B or D	√	/	√	Liu et al. (2012)
82	ESI‐	27.3	208.1076	208.1094	C_12_H_16_O_3_	5	−8.533	Beta asarone*	√	√	√	/
83	ESI‐	28.69	680.3809	680.3766	C_36_H_56_O_12_	9	6.219	Tenuifolin*	√	√	/	/

* Compounds confirmed by comparing with a reference standard.

### 3.2 GAPT can ameliorate scopolamine‐induced behavioral changes in learning and memory impaired mice

**Figure 2:**
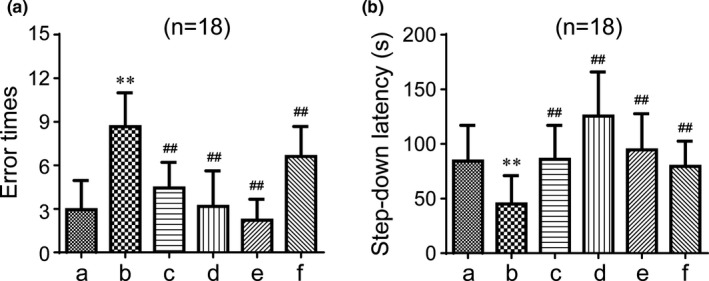
Effect of GAPT treatment on the error times (a) and latency (b) in the step‐down test after a half month of intragastric administration (*n* = 18). a, Control group; b, model group; c, donepezil group; d, GAPT high‐dose group; e, GAPT medium‐dose group; f, GAPT low‐dose group. The ET decreased and the SDL increased after donepezil and GAPT high‐dose, medium‐dose, and low‐dose treatment. **p < .01 versus control group, ^##^ *p* < .01 versus model group

**Figure 3:**
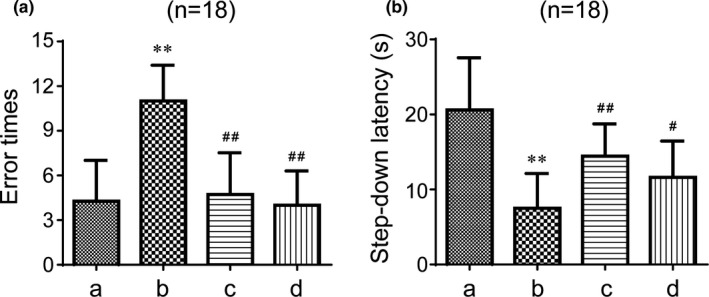
Effect of GAPT treatment on the error times (a) and latency (b) in the step‐down test after 1 month of intragastric administration (*n* = 18). a, Control group; b, model group; c, donepezil group; d, GAPT group. The ET decreased and the SDL increased after donepezil and GAPT treatment. **p < .01 versus control group, ^#^ *p* < .05, ^##^ *p* < .01 versus model group

Step‐down passive‐avoidance and Y maze tests were used to study the cognitive changes in mice after the intervention. The step‐down passive‐avoidance test is aimed to measure the ability to learn new information and to remember spatial location of laboratory animals by regularly offering a passive electrical stimulation. The results from the step‐down passive‐avoidance tests are shown in Figure 2. After half of a month of intragastric administration, the number of errors in the model group was increased compared to the control group (*p* < .01). The latency was shorter in the model group compared to the control group (*p* < .01). However, GAPT in any dose and donepezil significantly decreased error times and prolonged the step‐down latency (*p* < .01). These results show that GAPT can take effect after half a month of intragastric administration. To further observe the curative effect of GAPT, we choose a clinically effective dose of GAPT (the medium dose) and extended the time of administration to 1 month. As expected, the number of errors in the model group was increased compared to the control group (*p* < .01) and the latency was shorter in the model group (*p* < .01, Figure 3). Again, GAPT in medium dose and donepezil significantly decreased error times and prolonged the step‐down latency (*p* < .01 or *p* < .05, Figure 3).

**Figure 4:**
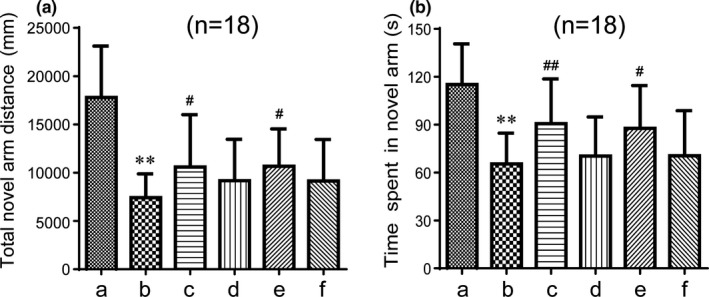
Effect of GAPT treatment on the total novel arm distance (a) and time spent (b) in the novel arm after a half‐month of intragastric administration (*n* = 18). a, Control group; b, model group; c, donepezil group; d, GAPT high‐dose group; e, GAPT medium‐dose group; f, GAPT low‐dose group. The distance and time spent of the GAPT medium‐dose group and donepezil group were significantly increased. **p < .01 versus control group, ^#^ *p* < .05, ^##^ *p* < .01 versus model group

**Figure 5:**
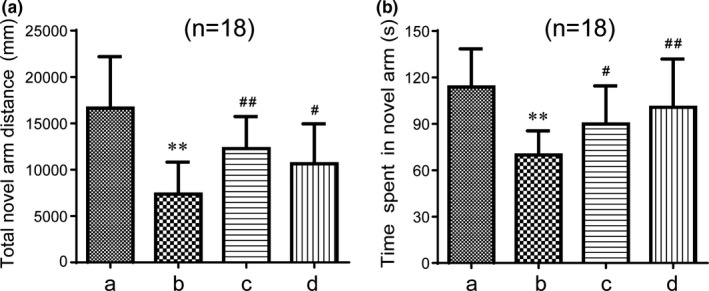
Effect of GAPT treatment on the total novel arm distance (a) and time spent (b) in the novel arm after 1 month of intragastric administration (*n* = 18). a, Control group; b, model group; c, donepezil group; d, GAPT group. The distance and time spent of the GAPT group and donepezil group were increased. **p < .01 versus control group, ^#^ *p* < .05, ^##^ *p* < .01 versus model group

The Y maze test is used to study the spatial recognition and memory ability of rodents, which are characterized by the natural habits of exploring new and different environments. The Y maze test can effectively reflect the ability of animals to recognize the new environment. Memory impaired mice usually spend less time and travel shorter distances while exploring the new arm. As shown in Figure 4, the total novel arm distance reordered in the Y maze was markedly shortened in the model group compared with the control group (*p* < .01). In contrast, the distance of the GAPT medium‐dose group and the donepezil group were longer than that of the model group (*p* < .05). The time spent in the novel arm was also shortened in the model group compared with the control (*p* < .01). The mice of GAPT medium‐dose group and the donepezil group also spent longer time in novel arm than the model group (*p* < .01 or *p* < .05). Although the distance and time in the new arm were extended in the high‐dose and low‐dose groups, there was no statistical significance. When administration for 1 month, In Figure 5, the total novel arm distance reordered and the time spent in the Y maze were markedly shortened in the model group compared to the control group (*p* < .01). Interesting, the GAPT medium‐dose group and the donepezil group significantly extend the distance and the time in the novel arm than the model group (*p* < .01 or *p* < .05).

### 3.3 GAPT can decrease the activity of MDA and AChE and increase the activity of Ach, ChAT, T‐SOD, and GPX in the brain of scopolamine‐treated mice

**Figure 6:**
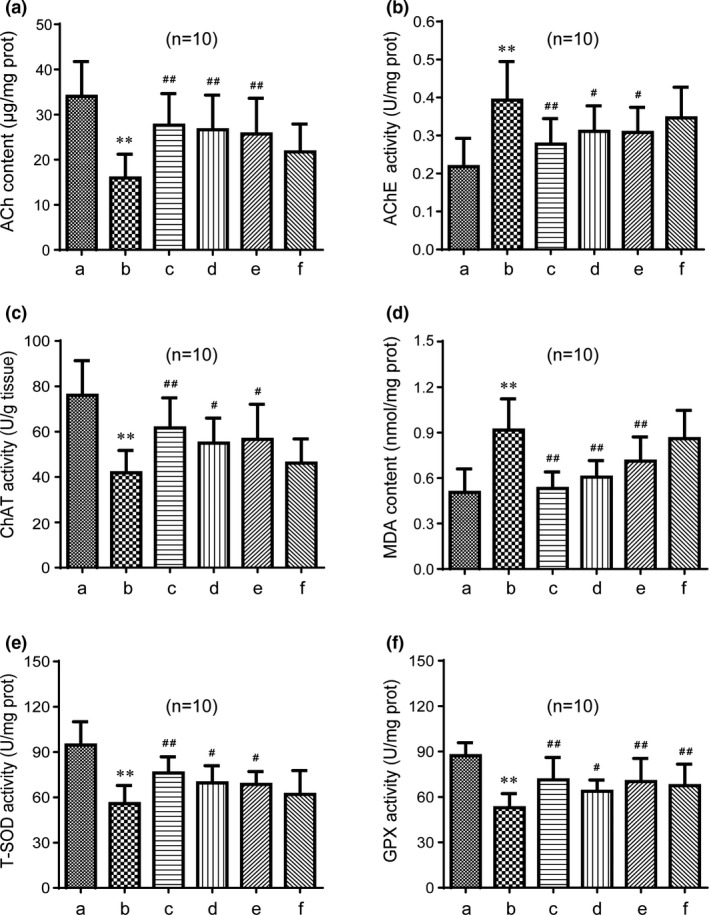
Effect of GAPT treatment on activities of ACh (a), AChE (b), ChAT (c), MDA (d), T‐SOD (e), and GPX (f) after a half month of intragastric administration (*n* = 10). a, Control group; b, model group; c, donepezil group; d, GAPT high‐dose group; e, GAPT medium‐dose group; f, GAPT low‐dose group. The activities of ACh, ChAT, GPX, and T‐SOD in the GAPT groups (large dose and medium dose) were increased, and the activities of AChE and MDA in the GAPT groups (large dose and medium dose) were reduced. The activity of GPX in the GAPT low‐dose group increased. **p < .01 versus control group, ^#^ *p* < .05, ^##^ *p* < .01 versus model group

**Figure 7:**
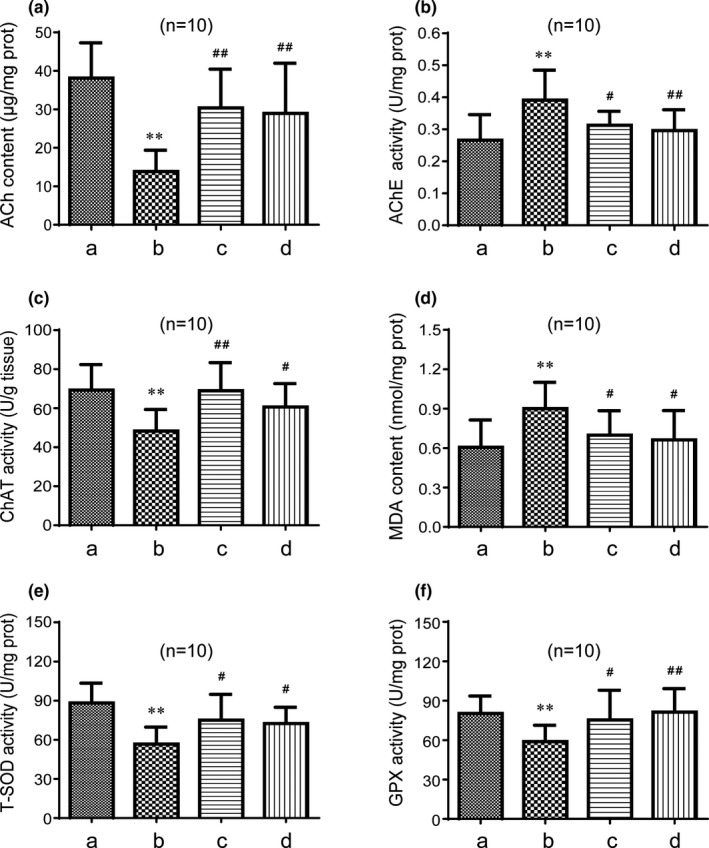
Effect of GAPT treatment on activities of ACh (a), AChE (b), ChAT (c), MDA (d), T‐SOD (e), and GPX (f) after 1 month of intragastric administration (*n* = 10). a, Control group; b, model group; c, donepezil group; d, GAPT group. The activities of ACh, ChAT, GPX, and T‐SOD in the GAPT group and donepezil group increased, and the activities of AChE and MDA in GAPT group and donepezil group reduced. **p < .01 versus control group, ^#^ *p* < .05, ^##^ *p* < .01 versus model group

As shown in Figures 6a and 7a, ACh content was significantly decreased by scopolamine (*p* < .01). Meanwhile, after half or 1 month of intragastric administration, donepezil remarkably (*p* < .01) increased the ACh content. Half month of GAPT intragastric administration can increase ACh content especially in the high‐ and medium‐dose groups (*p* < .01). One month of GAPT intragastric administration also can increase the ACh content (*p* < .01). As shown in Figures 6b and 7b, AChE activity was significantly enhanced by scopolamine (*p* < .01). Donepezil remarkably (*p* < .01 or *p* < .05) diminished the AChE activities after half or 1 month of administration. Half month administration of GAPT can diminish AChE activity especially in the high and medium dose groups (*p* < .05). One month administration of GAPT can also diminish the AChE activity (*p* < .01). As shown in Figures 6c and 7c, the ChAT activity was significantly diminished by scopolamine (*p* < .01). After half or 1 month of intragastric administration, donepezil remarkably (*p* < .01) increased the ChAT activities. Half month of GAPT administration can increase ChAT activity in the high‐ and medium‐dose groups (*p* < .05). One month administration of medium‐dose GAPT can increase the ChAT activity (*p* < .05). This result indicates that GAPT may play a neuroprotective role by inhibiting the decomposition and promote the synthesis of ACh in brain, thus protecting cholinergic neurons.

As shown in Figures 6d and 7d, MDA content was significantly increased by scopolamine (*p* < .01). Donepezil remarkably (*p* < .01 or *p* < .05) decreased the MDA content after half or 1 month of administration. Half month administration of GAPT can decrease MDA content in the high‐ and medium‐dose groups (*p* < .01). One month administration of GAPT can also decrease the MDA content (*p* < .05). As shown in Figures 6(e,f), and 7(e,f), the T‐SOD and GPX activities were significantly diminished by scopolamine (*p* < .01). After half or 1 month of administration, donepezil remarkably (*p* < .01 or *p* < .05) increased the T‐SOD and GPX activities. Half month of GAPT administration can increase T‐SOD and GPX activity in the high‐ and medium‐dose groups (*p* < .01 or *p* < .05). GAPT in low dose can also increase the GPX activity (*p* < .01). One month administration of medium‐dose GAPT can also increase the T‐SOD and GPX activities (*p* < .01 or *p* < .05). This result indicates that GAPT can play a neuroprotective role by reducing oxidative stress injury in the scopolamine‐induced memory impairment model.

### 3.4 GAPT can decrease expression of AChE and increase expression of ChAT, GPX1, and SOD1 in hippocampus and basal forebrain of scopolamine‐treated mice

**Figure 8:**
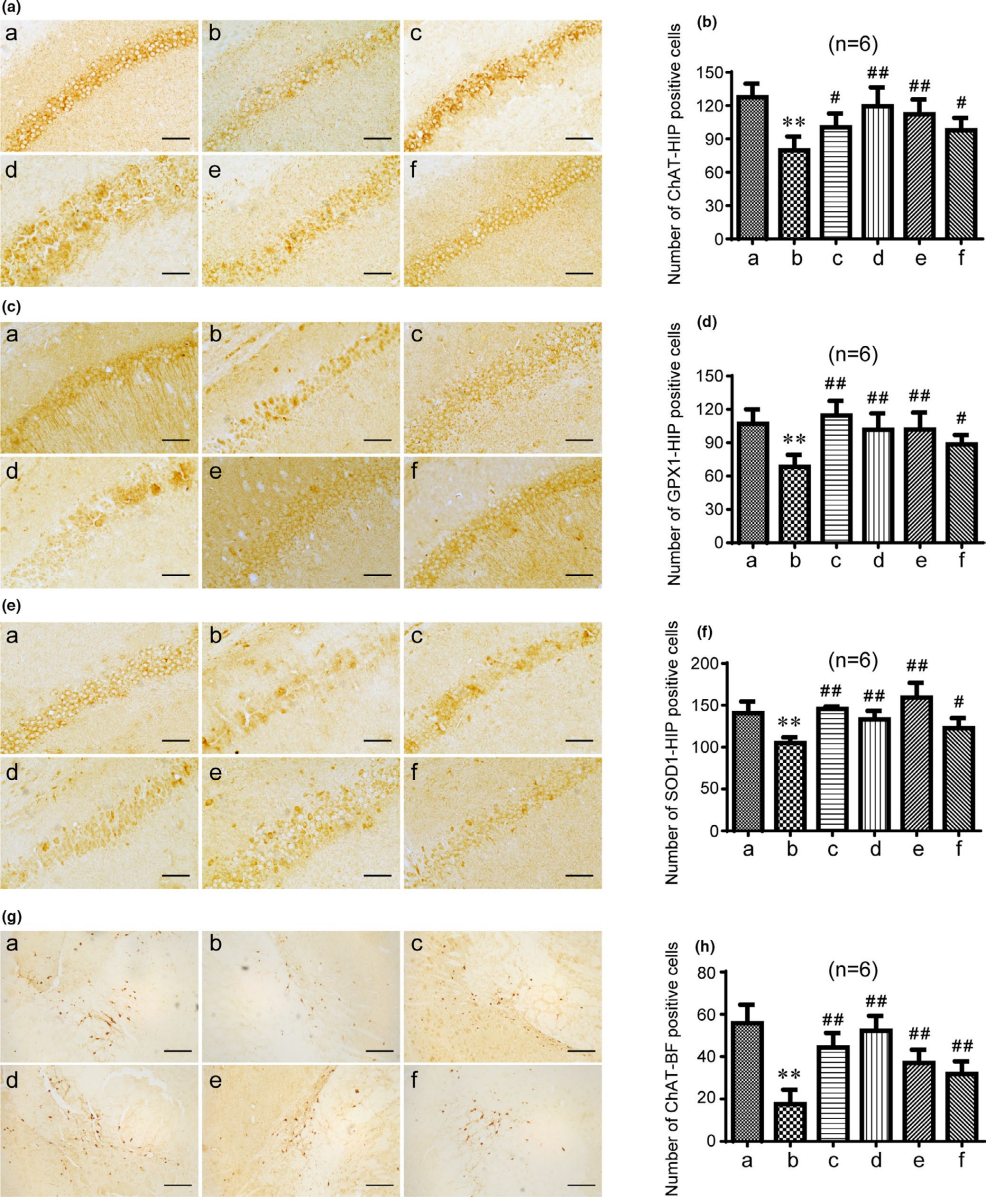
Effect of GAPT treatment on distribution and positive cells of ChAT (a, b), GPX1 (c, d), and SOD1 (e, f) in the CA1 region of the mouse hippocampus and ChAT (g, h) in basal forebrain after a half‐month of intragastric administration (*n* = 6). HIP, hippocampus; BF, basal forebrain; a, control group; b, model group; c, donepezil group; d, GAPT high‐dose group; e, GAPT medium‐dose group; f, GAPT low‐dose group. The positive cells of ChAT, GPX1, and SOD1 in the GAPT groups (high dose, medium dose, and low dose) and donepezil group increased. **p < .01 versus control group, ^#^ *p* < .05, ^##^ *p* < .01 versus model group. a, c, e, scale bar = 50 μm; g, scale bar = 200 μm

**Figure 9:**
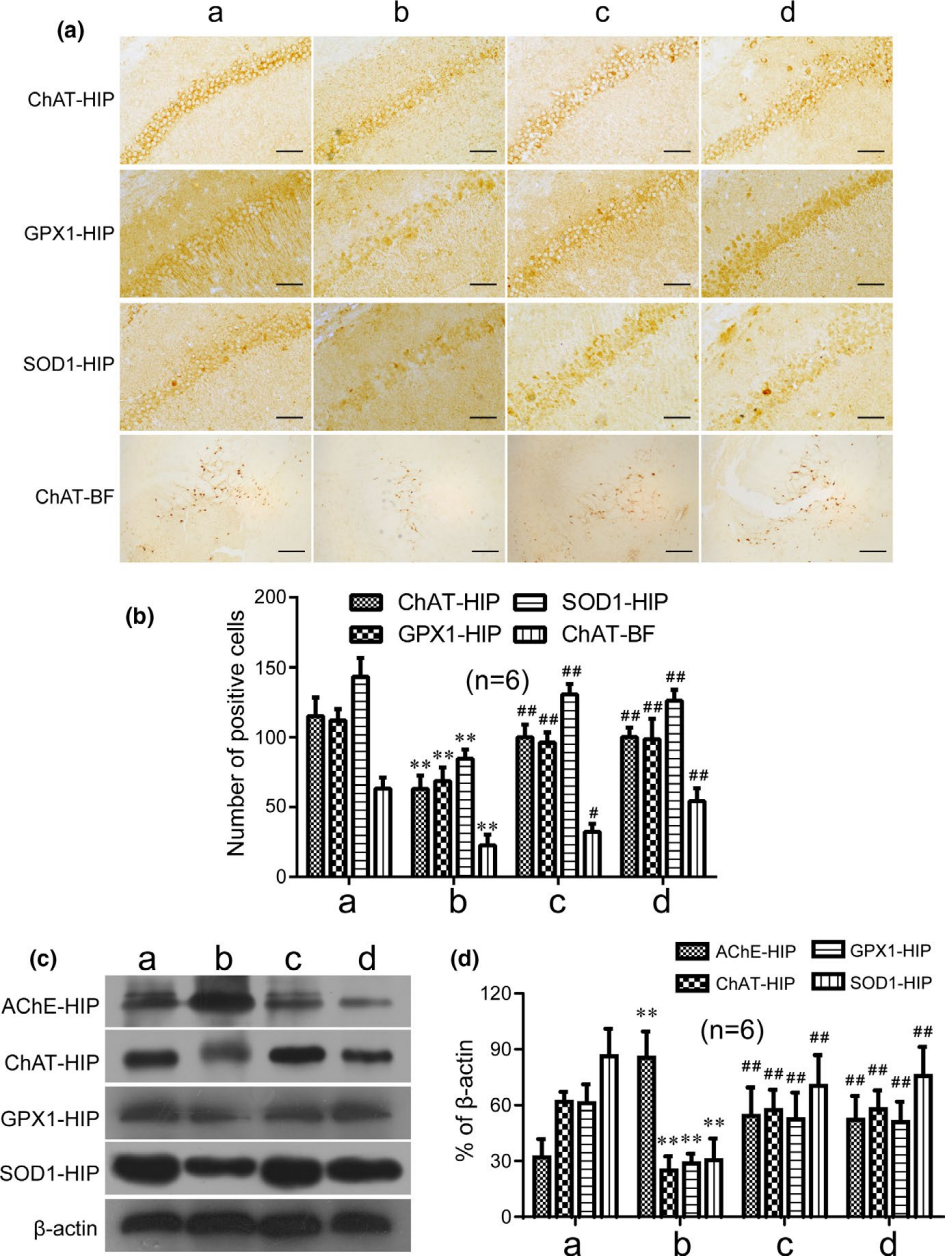
(a, b) Effect of GAPT treatment on distribution and positive cells of ChAT, GPX1, and SOD1 in the CA1 region of the mouse hippocampus or basal forebrain and (c, d) protein expression level of AChE, ChAT, GPX1, and SOD1 after 1 month of intragastric administration (*n* = 6). HIP, hippocampus; BF, basal forebrain; a, control group; b, model group; c, donepezil group; d, GAPT group. The positive cells and protein expression level of ChAT, GPX1, and SOD1 in the GAPT group and donepezil group increased. The GAPT group and donepezil group decreased the expression of AChE. **p < .01 versus control group, ^##^ *p* < .01 versus model group. a, ChAT‐HIP, GPX1‐HIP, SOD1‐HIP, scale bar = 50 μm; a, ChAT‐BF, scale bar = 200 μm

As shown in Figure 8(a‐f), the positive cells of ChAT, GPX1, and SOD1 were significantly reduced by scopolamine (*p* < .01). GAPT in any dose and donepezil remarkably (*p* < .01 or *p* < .05) increased the positive cells after half a month gavage in hippocampus. In the meantime, the positive cells and relative protein expression levels of ChAT, GPX1, and SOD1 were significantly decreased by scopolamine (*p* < .01), which significantly reversed (*p* < .01) after 1 month gavage of medium‐dose GAPT and donepezil in hippocampus (Figure 9(a‐d)). Consistent with previous studies, scopolamine can increase the protein expression of AChE (*p* < .01). Not surprisingly, medium‐dose GAPT and donepezil can decrease the protein expression of AChE in hippocampus (*p* < .01, Figure 9(c,d)).

Acetylcholine signals in both the hippocampus and cortex are mainly originated from the basal forebrain projection (Ballinger, Ananth, Talmage, & Role, 2016). In order to further confirm the role of GAPT in cholinergic pathways, we measured the ChAT expression in basal forebrain. As shown in Figure 8(g,h), the positive cells of ChAT was significantly reduced by scopolamine (*p* < .01). GAPT in any dose and donepezil remarkably (*p* < .01) increased the positive cells after half a month gavage in basal forebrain. The positive cells of ChAT were significantly decreased by scopolamine (*p* < .01), which significantly reversed (*p* < .01 or *p* < .05) after 1 month gavage of medium‐dose GAPT and donepezil in basal forebrain (Figure 9(a,b)).

## 4 DISCUSSION

Numerous lines of evidence show that scopolamine is capable of blocking cholinergic neurotransmission. This ability has led scopolamine to be widely employed to induce AD‐like pathology in vivo and in vitro. Scopolamine can impair the processes of learning acquisition and consolidation (More, Kumar, Cho, Yun, & Choi, 2016), significantly reduce Ach activities, and increase oxidative stress in the hippocampus and prefrontal cortex in mice. Experimental data confirm that exposure to scopolamine (1–3 mM) can significantly decrease human neuroblastoma SH‐SY5Y cell viability (Puangmalai et al., 2017) and induce PC12 cell mitochondrial and plasma membrane damage (Pandareesh & Anand, 2013). To investigate the particular structures related to memory and learning, scopolamine can also be used (Newman et al., 2017). Improved acetylcholine function recognized as an important way to improve memory. Therefore, in this study, scopolamine was employed to observe whether GAPT can enhance or protect the stability of cognitive activity.

The early research indicates that GAPT can decrease the expression level of endogenous Aβ peptide by inhibiting the PS1 activity in APPV717I transgenic mice (Tian et al., 2009). Eight months and three months after administration with GAPT, spatial learning function and memory abilities were significantly enhanced, suggesting that GAPT could prevent cognitive impairments and protect learning and memory function in an AD‐like rat model (Tian et al., 2006). However, whether GAPT could ameliorate the scopolamine‐induced memory impairment and the latency before GAPT becomes effective are still unclear. In our experiment of SDPA, after a half month of intragastric administration, donepezil and GAPT in any dose indeed decreased the number of errors and extended the latency, indicating that GAPT has a certain effect on memory acquisition and reproducing ability in scopolamine‐induced AD‐like mice. When the gavage time was extended to 1 month, the medium dose of GAPT can also reduce the number of errors and prolonged the latency and has the same efficacy as donepezil in the treatment of scopolamine‐induced AD‐like mice. In our experiment of Y maze test, donepezil and GAPT can prolong exploring time and distance in the new arm. It is noteworthy that in the subsequent 1‐month experiment, donepezil and GAPT (medium dose) can prolong exploring time and distance in the new arm, hence improving spatial recognition capability.

Ach plays an important role in the central nervous system. Ach is the central neurotransmitter that most closely relates to learning and memory processes. Acetyl‐CoA and choline participate in Ach synthesis because of the catalysis of acetyltransferase (ChAT). AD patients usually suffer the loss of cholinergic neurons in the cerebral hippocampus and cortex (Schliebs & Arendt, 2006) along with decreased cholinergic activity, which is possibly due to increased activity of AChE (Khan, 2009). Severely diminished ChAT expression was also observed (Orta‐Salazar, Aguilar‐Vázquez, et al., 2014) in this process. Some studies also suggested that oxidative stress was closely related to the increase of AChE activity (Inestrosa, Dinamarca, & Alvarez, 2008). In this study, donepezil and GAPT can decrease AChE activity and protein expression level and increase the activity of ChAT and ACh in scopolamine‐treated mice. Scopolamine‐induced learning and memory disorder was effectively mitigated by GAPT, thereby protecting the cholinergic system and maintaining the normal activity of ChAT and AChE. However, we cannot rule out the possibility of AchR receptor agonist in GAPT compound at present, and we hope to do this work at next step.

The imbalance between pro‐oxidant stress and antioxidants frequently leads to oxidative stress. High metabolic rates make the brain the most sensitive organ to hypoxia, and the brain is particularly susceptible to oxidative stress‐mediated damage (Butterfield, Drake, Pocernich, & Castegna, 2001). Some researchers are convinced that oxidative damage plays a large part in the initial process of AD (Arimon et al., 2015; Manoharan et al., 2016). Previous studies have highlighted that oxidative stress leads to the accumulation of amyloid β42 (Misonou, Morishima‐Kawashima, & Ihara, 2000) and mitochondrial dysfunction (Moreira, Carvalho, Zhu, Smith, & Perry, 2010; Onyango, Dennis, & Khan, 2016; Wong‐Guerra et al., 2017). Fortunately, antioxidant enzymes, including GPX and SOD, can protect tissues against reactive oxygen species (ROS) (Pohanka, 2014). Our results show that donepezil and GAPT markedly increased SOD activity and GPX activity in the brains of scopolamine‐treated mice, indicating that GAPT could improve oxidative stress impairment.

In our previous studies, we report that GAPT has a neuroprotective effect over 3 months of intragastric administration. In this study, we first found that both short‐term (half a month) and long‐term (1 month) of intragastric administration of GAPT could relieve the effects induced by scopolamine. To confirm the real effects of GAPT in neurons and brain regions after half a month of intragastric administration of GAPT, an LC‐MS method was established to study the chemical compounds and in vivo metabolites of GAPT. Of the 83 compounds identified in GAPT, 42 compounds were able to enter the blood, and, surprisingly, 43 compounds might pass through BBB and reach the specific brain areas or neurons. At least, we can sure that echinacoside, salvianolic acid A, ginsenoside Rb1, ginsenoside Rg2, pachymic acid, and beta asarone which identified by comparing with reference standards could be absorbed into mice brain. These findings provided informative groundwork for further pharmacokinetic studies of GAPT prescription.

In conclusion, GAPT can ameliorate the scopolamine‐induced behavioral changes in learning‐ and memory‐impaired mice. GAPT reduced the hydrolysis of ACh by reducing the activity and protein expression of AChE. At the same time, it increased the synthesis of ACh by increasing the activity, protein expression, and distribution of ChAT, thus improving the cholinergic nerve function. Meanwhile, GAPT increases the activity, protein distribution, and expression of SOD1 and GPX1, reduces the damage of ROS to cells, improves the damage caused by oxidative stress, and plays a neuroprotective role. Both short term and long term of intragastric administration of GAPT can improve the learning and memory ability of scopolamine‐induced memory impairment model mice, and its mechanism is related to protecting cholinergic neurons and reducing oxidative stress injury.

## CONFLICT OF INTEREST

The authors declare that there are no competing interests associated with the manuscript.

## AUTHOR CONTRIBUTIONS

Zhenhong Liu, Gaofeng Qin, and Lulu Mana performed the experiments, analysis and interpretation of the data, and wrote the manuscript. Yunfang Dong, Shuaiyang Huang, Yahan Wang, Yiqiong Wu, Jing Shi, and Jinzhou Tian participated in experiments and result analysis. Pengwen Wang was responsible for experimental design and fund support and approved the final version for publication.

## Data Availability

The data that support the findings of this study are available from the corresponding author upon reasonable request.
